# Improving the biological interfacing capability of diketopyrrolopyrrole polymers *via* p-type doping†

**DOI:** 10.1039/d3tc01148h

**Published:** 2023-05-11

**Authors:** Ryan P. Trueman, Peter Gilhooly Finn, Megan M. Westwood, Avishek Dey, Robert Palgrave, Alethea Tabor, James B. Phillips, Bob C. Schroeder

**Affiliations:** a Center for Nerve Engineering, UCL London UK; b Department of Pharmacology, UCL School of Pharmacy, University College London London UK; c Department of Chemistry, University College London London UK b.c.schroeder@ucl.ac.uk

## Abstract

Polydiketopyrrolopyrrole terthiophene (DPP3T) is an organic semiconducting polymer that has been widely investigated as the active layer within organic electronic devices, such as photovoltaics and bioelectronic sensors. To facilitate interfacing between biological systems and organic semiconductors it is crucial to tune the material properties to support not only cell adhesion, but also proliferation and growth. Herein, we highlight the potential of molecular doping to judiciously modulate the surface properties of DPP3T and investigate the effects on Schwann cell behaviour on the surface. By using p-type dopants FeCl_3_ and Magic Blue, we successfully alter the topography of DPP3T thin films, which in turn alters cell behaviour of a Schwann cell line on the surfaces of the films over the course of 48 hours. Cell numbers are significantly increased within both DPP3T doped films, as well as cells possessing larger, more spread out morphology indicated by cell size and shape analysis. Furthermore, the viability of the Schwann cells seeded on the surfaces of the films was not significantly lowered. The use of dopants for influencing cell behaviour on semiconducting polymers holds great promise for improving the cell-device interface, potentially allowing better integration of cells and devices at the initial time of introduction to a biological environment.

## Abstract 2

Polydiketopyrrolopyrrole terthiophene (DPP3T), a high-performing conjugated polymer, holds great potential as active material for bioelectronics. Herein, its surface properties are modulated through p-type doping, thereby enhancing the cell behaviour on top of the doped films.

## Graphical abstract

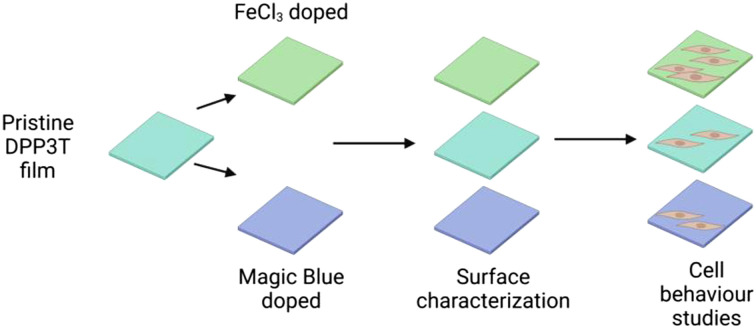

## Introduction

Cell adhesion is a critical biological process that plays a vital role in various physiological and pathological events such as tissue formation, wound healing, and organ development. The ability to modulate cell adhesion using engineered biomaterials holds significant promise within the fields of regenerative medicine and tissue engineering. In recent years, the development of materials with controlled surface properties and topographies has opened new avenues to manipulate cell adhesion and ultimately influence cell behaviour.^1–3^ In this context, the application of conjugated polymers, such as donor–acceptor conjugated molecules, has shown promise in achieving tuneable surface properties,^4,5^ but these donor–acceptor polymers have primarily been used for creating devices, such as organic electrochemical transistors (OECTs), rather than used as biomaterials for regenerative medicine applications. However, what has been studied is the use of conductive polymers such as polypyrrole (PPy) and poly(3,4-ethylenedioxythiophene)–poly(styrenesulfonate) (PEDOT:PSS) for cellular interfacing^6–8^ and their application for both electrical stimulation as an active option, and controlled surface properties for a passive benefit to regenerating cells.^9^ Furthermore, conductive polymers such as PPy and PEDOT:PSS are inherently doped during the synthesis, therefore by controlling the degree of doping, it may be possible to tune the surface properties to optimise interactions between the conducting polymers and biological systems.

**Scheme 1:**
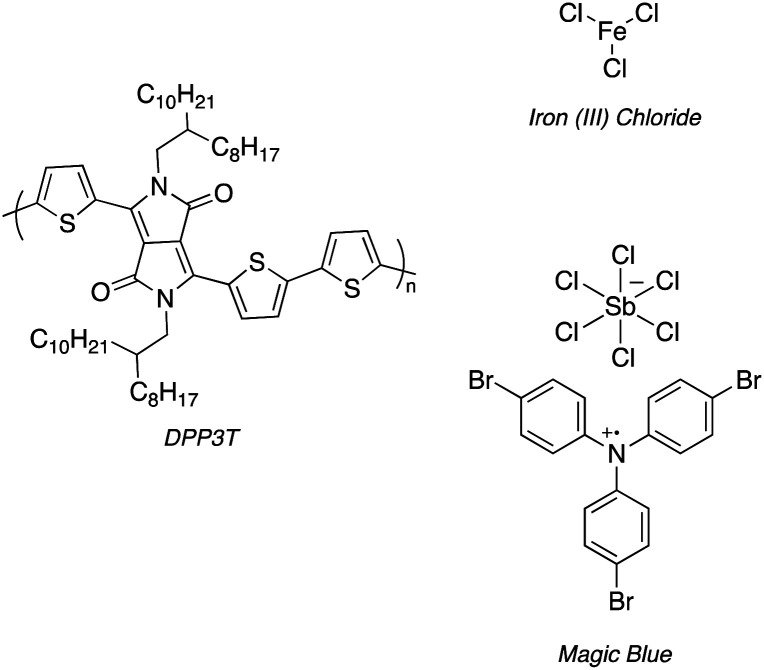
Structure of DPP3T and 2 dopants, iron(iii) chloride (FeCl_3_) and Magic Blue investigated within this experimental report.

Poly(diketopyrrolopyrrole terthiophene) (DPP3T) depicted in Scheme 1 has emerged as a promising organic semiconducting polymer with high charge carrier mobility and excellent processability, and may find use within bioelectronic applications as an engineered biomaterial.^10,11^ However, the standard DPP3T polymers within the literature possess branched alkyl chains, which are ideal for aiding solubility during material processing, yet are not optimal substrates for influencing biomaterial–cell interfacing.^12,13^

One strategy is to alter the side chains of the polymer to improve cell adhesion and proliferation,^14–16^ for example through the addition of poly lysine-based side chains.^17^ Another alterative, albeit not often explored, is through introducing dopants to the polymers.^18^ Doping DPP3T can alter not only the electronic structure, but also the surface properties of the thin film, potentially enhancing cell adhesion and proliferation. Two commonly used p-type dopants for doping organic semiconducting polymers are iron(iii) chloride (FeCl_3_) and Magic Blue.^19,20^

FeCl_3_ and Magic Blue are both potent oxidants for DPP3T, and numerous studies have demonstrated the dramatic increase in conductivity in doped DPP3T films compared to pristine ones.^19,21^ The doping mechanism varies between the organic semiconducting material, with polymer backbone, sidechain functionalities and processing method all playing a role in the interaction between polymer and dopant. In depth discussion of doping on the molecular level is out of the scope of this experimental report, and the reader is directed towards relevant reviews for a more in-depth account on the current understanding of doping within organic semiconductors.^22–24^

In our study, we attempted to dope DPP3T thin films using sequential doping methods with the three separate dopants of differing electron affinities,^25^ namely iron(iii) tosylate hexahydrate (Fe(Tos)_3_·H_2_O), anhydrous FeCl_3_ and Magic Blue. Cellular adhesion favours either roughened, higher surface energy or charged surfaces. Charged surfaces in particular are known to provide additional interactions with oppositely charged cell adhesion proteins.^26^ Therefore, we investigated the use of p-type dopants to alter the surface properties and charge of DPP3T to influence cell adhesion and viability.

We first sought to assess the doping potential of these dopants in air, and characterize the surfaces of the doped thin films, using X-photoelectron spectroscopy (XPS), atomic force microscopy (AFM) and water contact angle to assess if doping had the potential to alter cell adhesion of Schwann cells *in vitro*. Lastly, we undertook preliminary assays to investigate the use of the doped films as a novel biomaterial through cell behaviour and viability assays. The potential degradation of the doped DPP3T films was also studied over the cell culture assay period.

## Results and discussion

### p-type doping of DPP3T thin films

UV-Vis-NIR spectroscopy was used as an initial study to not only evaluate the optical properties of the neutral and doped thin films (Suppl. 1, ESI†), but also to establish the minimum dopant concentration to maximise charge transfer. A decrease in intensity of the neutral DPP3T absorption peak (772 nm) and appearance of the polaron peaks (≈1250 nm and >2000 nm) shows that charge transfer has occurred from DPP3T to the dopant and is proportional to the polaron concentration. The higher intensity of the polaron peaks also qualitatively indicates more effective charge transfer. We doped the neutral DPP3T films *via* depositing dopant solution in acetonitrile (ACN) onto the film and left to soak for 60 seconds, then the excess solution was removed by spinning at high speed (2000 rpm) on a spin coater. While spinning, the films were washed with ACN to remove any excess and unreacted dopant on the surface. Firstly, we doped using iron(iii) tosylate hexahydrate (Suppl. 1, ESI†). However, the iron(iii) tosylate failed to alter the electronic properties DPP3T thin film and we excluded this dopant for the remainder of the study. This could either be due to iron(iii) tosylates inability to mix effectively with the pristine DPP3T thin film, or the hexahydrated ion being a weaker oxidant not suitable to initiate charge transfer due to a poor energetic offset.^27^

**Fig. 1:**
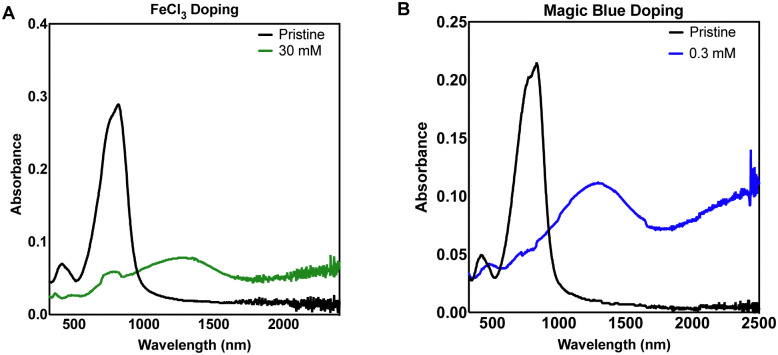
Thin film UV-Vis-NIR absorbance spectra of sequentially doped thin films of DPP3T using (A) FeCl_3_ and (B) Magic Blue as dopants.

Next, we utilized a commonly reported p-type dopant for organic semiconducting donor acceptor polymers, anhydrous FeCl_3_^28,29^ (Fig. 1(A)). Using a 30 mM solution of FeCl_3_ in ACN, we observed bleaching of the neutral transition and appearance of the polaron peaks attributed to charge transfer between DPP3T and FeCl_3_. Most studies are carried out using a glovebox with a nitrogen atmosphere, so differences in concentrations between our dopant and the literature are difficult to compare, however doping in air was appropriate for this experiment, as the study was focused on investigating cell adhesion under biological conditions.

We expanded our study to Magic Blue (Fig. 1(B)), a strong p-type dopant recently employed successfully with DPP3T polymers.^19^ Using the same doping method, Magic Blue required a 100-fold lower concentration of 0.30 mM to completely bleach the neutral peak of pristine DPP3T at ≈900 nm, whereas with the FeCl_3_ doped films, we failed to completely bleach the peak at our highest concentration of 30 mM FeCl_3_ (Suppl. 1, ESI†). 60 mM was required to completely bleach the neutral DPP3T peak, however the overall doping was not considerably greater, when using double concentration of the FeCl_3_.

For the purposes of testing our hypothesis of modulating surface properties of organic semiconducting polymers, the doping levels were not intended to be equal between the dopants. The concentrations chosen were based upon toxicity literature for FeCl_3_ and *in vitro* cell cultures.^30,31^ FeCl_3_ is known to be cytotoxic so caution was drawn around choosing the highest doping level, therefore the 30 mM sample was chosen as it still provided significant doping to the DPP3T polymer. With the DPP3T doped with Magic Blue, we found that only a very low concentration of 0.3 mM was needed to completely bleach the neutral DPP3T absorption peak and deemed it unjustified to explore higher dopant concentrations, especially considering the potentially toxic side effects of antimony. The specific toxicity of Magic Blue is unknown due to a lack of studies investigating its effects on cell cultures, yet considering the relevant literature on antimony toxicity, likely complex. Antinomy is reported to have toxic side effects within patients of cardiotoxicity and pancreatitis,^32^ yet at the same time is used as a therapeutic for the treatment of two parasitic diseases, leishmaniasis and schistosomiasis.^33,34^

For the following characterization and cell experiments, we took forward the 30 mM FeCl_3_ and 0.30 mM Magic Blue concentrations. Because the primary aim of this study was to investigate the effect of different dopants on surface morphology and cell adhesion, and not to maximise the electronic properties of the polymer.

We then studied the composition of elements on the surface of the neutral and doped films using XPS (Suppl. 2 and 3, ESI†). In the survey scan, the neutral DPP3T films show peaks associated to oxygen, nitrogen, carbon and sulphur arising from the polymer. FeCl_3_ doped films showed the same peaks as the neutral films however, peaks for Fe 2p and Cl 2p are also present indicating FeCl_3_ is on the surface. Magic Blue doped films also tell a similar story, but the new peaks arise from Sb 3d_3/2_ around 540 eV and Cl 2p. The Sb 3d_5/2_ overlaps with O 1s. Interestingly we observed no bromine present in the survey scan of the Magic Blue doped films indicating that the tris(4-bromophenyl)ammoniumyl counter cation is no longer present possibly due to the washing step.^35^ We also found that the S 2p and N 1s peaks for the MB and FeCl_3_ doped DPP3T films shift to higher binding energies by around 2 eV compared with the undoped film, which has been associated to oxidation of the polymer in the literature.^36^

### Morphology of the doped films

**Fig. 2:**
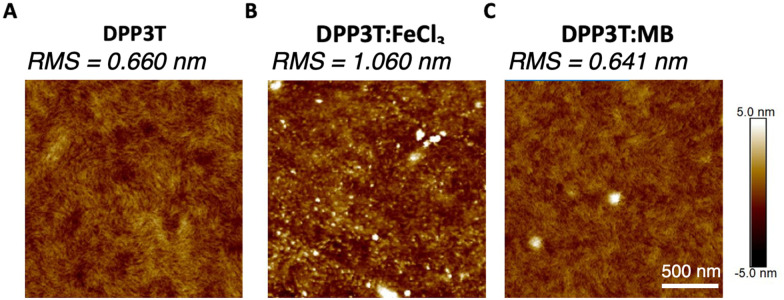
AFM images of the DPP3T (A) pristine film surface, (B) doped with FeCl_3_ and (C) doped with Magic Blue with RMS values of each of the films.

The morphology of the pristine DPP3T and films doped with FeCl_3_ and Magic Blue were investigated, because in biomaterials the surface roughness and topology play a crucial role in cell adhesion within biological systems.^24,37^ Atomic force microscopy (AFM) was utilized to gain insight into the nanoscale differences within the topology of the different films (Fig. 2 and Suppl. 4, ESI†). The pristine DPP3T film (Fig. 2(A)) presented a homogenous surface, with a route mean squared roughness (RMS) of 0.660 nm and swirl like regions. The FeCl_3_ doped DPP3T thin film on the other hand presented a considerably rougher topology (RMS = 1.06 nm), and the swirl like pattern seen within the pristine film was no longer visible (Fig. 2(B)). There were also features that were considerably higher than the plane of the film, which we attribute to dopant aggregates on the surface. Interestingly, the Magic Blue doped DPP3T thin film presented very similar topology to that of the pristine film, including the swirl like pattern (Fig. 2(C)) and a lower overall roughness (RMS = 0.641 nm). Like the FeCl_3_ doped film, the Magic Blue doped sample also possessed a small number of raised features.

FeCl_3_, a widely used dopant for DPP based polymers has shown to alter the crystalline microstructure within the solid state and incorporating high concentrations (*i.e.* 30 mM) have been shown to increase film roughness.^36,38–40^ On the other hand, the structural lattice changes induced by doping DPP based polymers with Magic Blue have not been widely studied. Zhong *et al.* recently showed that Magic Blue intercalates into the amorphous regions of highly oriented thin films of poly(3-hexylthiophene) (P3HT) upon sequential doping, leading to no change in the structural reorganisation within the π-stacking direction.^41^ Whilst we cannot exclude that some of the topological differences observed in Fig. 2 arise from the different intercalations of FeCl_3_ and Magic Blue within the polymer microstructure, we ascribe the changes observed primarily to the different amounts of dopant introduced into the films.

To understand the hydrophilicity of the doped polymer films in comparison to the pristine, water contact angle measurements and surface energy calculations were performed (Suppl. 5, ESI†). The contact angles of two water droplets were measured for each film and on average the FeCl_3_ doped films exhibited a slightly lower contact angle (95°) than pristine DPP3T (98°) and DPP:Magic Blue (101°). Measuring the contact angle of 2 diiodomethane droplets as well for each film, we could then calculate the surface energy of the films. Although we found that the associated energies are very similar (∼36 mJ m^−2^) and probably within the error of the measurement, FeCl_3_ doped films do exhibit the highest surface energy and the lowest water contact angle (Suppl. 5 and Tables 1, 2, ESI†), indicating a more hydrophilic surface which may influence cell adhesion properties.^37,42^

### Behaviour of Schwann cells on top of doped films

**Fig. 3:**
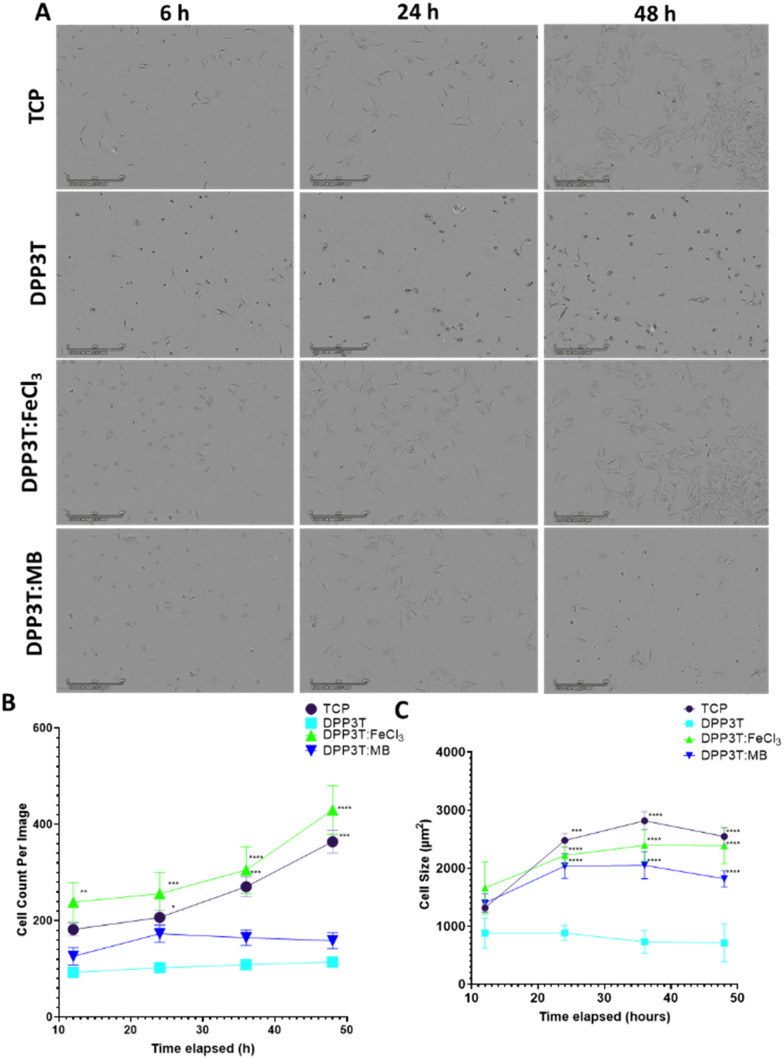
Behaviour of Schwann cells seeded on top of the DPP3T thin films. (A) Phase contrast microscopy at 6 hours, 24 hours and 48 hours post cell seeding on top of the fibres. (B) Quantification of the number of cells on top of each film per field of view over time. Field of View = 1.7 mm^2^ (C) Cell size over the time course of the assay. *N* = 6 independent DPP3T films per experimental group, with 9 images per film, per time point analysed for the cell count per image mean. Data presented as mean ± SEM. (C) Stats = Two-Way ANOVA (B, *p* = 0.0127, C, *p* = <0.0001) with Dunnett's multiple comparisons test run *post hoc* against the pristine DPP3T film. *p* < 0.05 denotes significance, with * = *p* < 0.05, ** = *p* < 0.01, *** = *p* < 0.001 **** = *p* < 0.0001 denoted on the graphs.

After characterising the surface morphology of pristine DPP3T thin films and their doped counterparts surface morphology and hydrophobicity, the effects of doping the films on cell growth and survival were investigated. Because our research focusses on neural interfacing applications of organic electronics, we seeded SCL1.4/F7 rat Schwann cells on top of the thin films cast on glass coverslips and then imaged over the course of 48 hours to monitor changes in the number of adherent cells. Phase contrast microscopy was used to visualize cells on the thin films (Fig. 3(A)).

Cell population changes over the 48 hours possessed similar kinetics between the tissue culture plastic (TCP) and the FeCl_3_ doped DPP3T (Fig. 3(B)). TCP was used a positive control, as this was plasma treated polystyrene and represents a model material for cell adhesion widely used within cell culture.

After 48 hours in culture, significantly more cells were observed per field of view on both the control TCP (3.2-fold increase in cell number, *p* = 0.0009) and FeCl_3_ doped DPP3T thin films (3.8-fold increase in cell number, *p* = 0.0001) in comparison to the pristine DPP3T film. There was no significant difference between the pristine DPP3T and the Magic Blue doped film, despite the slight increase in cell number at 48 hours (1.4-fold increase in cell number, *p* = 0.6061).

On TCP, the seeded cells possess wide, flattened morphology which is typical of a cell that is adhered to a flat surface.^43,44^ A method of characterising cellular behaviour on thin films is to use cell size as a metric to indicate cell adhesion and spreading (Fig. 3(C)). Using the Sartorius Incucyte cell-by-cell analysis, it was possible to measure cells within the field of view for size (Fig. 3(C)) and cell shape (Suppl. 6, ESI†). At the end of the 48 hour assay, the cell size on doped materials was significantly different in comparison to the undoped, DPP3T pristine film (714.2 ± 325.37 μm mean size ± Std. Dev.). The FeCl_3_ doped films had cells that were 3.3-fold larger than those seeded on the pristine film (*p* = 0.0001) and the Magic Blue doped films possessed cells that were 2.5 fold larger in comparison to the pristine DPP3T film. Cells seeded on the positive TCP control were of similar size to the FeCl_3_ doped films. Cells seeded on the smoother, non-doped pristine DPP3T surface were both smaller and lower in number than the two doped films and the TCP, indicating a potential difficulty adhering to the surface.

Within the pristine DPP3T thin film and two doped films, the cells possessed a visually different morphology (Fig. 3(A)). The change in morphology was quantified using cell-by-cell image analysis from the phase contrast images. This allows assessment of cell shape, in particular eccentricity, with values ranging from 0, representing a perfect circle, to 1, constituting an elongated shape. This information can be combined with information about cell size to give insights into the overall adhesion of the cells on top of the respective surface.

The cells on DPP3T possessed ellipsoidal morphology (cell eccentricity = 0.67), which is an indication that the cells were less able to elongate on the undoped conjugated polymer surface. However, upon doping the film with FeCl_3_, the cells adopt a comparable morphology to those deposited on TCP (0.79 and 0.78, respectively), with the cells flattened and spread across the surface of the doped thin film. The sample doped with Magic Blue showed similar characteristics to the pristine DPP3T film with regards to cell number per image on the surface of the film, however the cell shape showed an eccentricity of 0.79, which is comparable to the parameters observed for the TCP and FeCl_3_ doped film (Suppl. 6, ESI†). The reduction in total cell number may have been due to the potentially toxic antinomy salt that may have remained after the initial doping.^45^

### Cell viability after 48 hours of culture

**Fig. 4:**
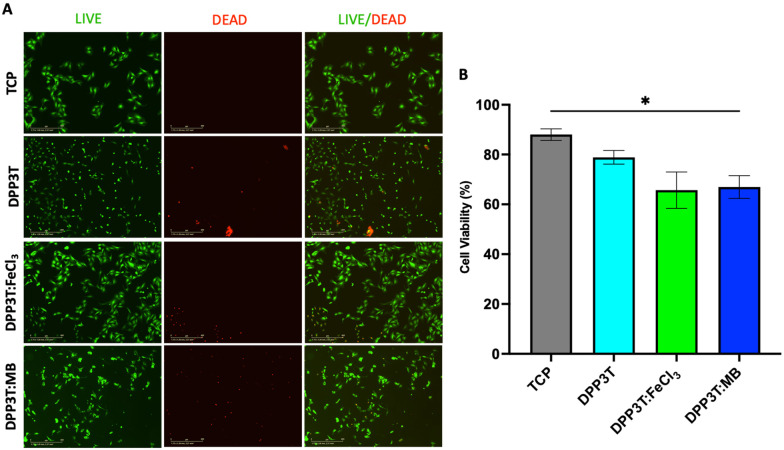
Viability of Schwann cells seeded on top of DPP3T thin films. (A) Immunofluorescence of SCL1.4/F7 Schwann cell 48 hours seeding on top of the DPP3T thin films. Live (green): calcein-AM, dead (red): ethidium homodimer-1. (B) Quantification of cell viability from the live/dead stain. *N* = 6 independent films, with 9 images analysed per film. Stats = One Way ANOVA and Tukey's multiple comparisons *post hoc* test. * = *p* < 0.05.

After investigating the behaviour of Schwann cells seeded on the different surfaces, we sought to evaluate cell viability after 48 hours of growth on the various surfaces (Fig. 4). In line with the previous experiments, TCP was used as positive control, and doped materials were compared with the pristine DPP3T thin film. Using immunofluorescence microscopy, it was possible to visualize the morphology of the cells (Fig. 4(A)). The number of live cells was calculated using the calcein-AM cytosolic stain and dead cells were identified with the ethidium homodimer-1 nuclei stain to quantify cell viability on the surfaces of the thin films (Fig. 4(B)).

The TCP group unsurprisingly possessed the highest percentage of live cells (88.02% ± 4.03% viability, mean and Std. Dev.), with the pristine DPP3T film supporting cell viability (78.87% ± 9.51% viability, mean ± Std. Dev.). This highlights that the organic polymer, and the processing method of spin coating from harmful chlorinated organic solvents does not result in significantly increased cell cytotoxicity. With regards to the FeCl_3_ and Magic Blue doped DPP3T thin films, there was lower mean cell viability in comparison to the pristine film, however this was found to not be statistically significant (Fig. 4(B)) (88.02% mean for the pristine film, 65.68% ± 12.65% and 66.95% ± 11.23%; *p* = 0.1428 & 0.0733, mean and Std. Dev., FeCl_3_ and Magic Blue respectively). There was however significantly lower cell viability on the Magic Blue doped surface in comparison to the TCP group (*p* = 0.0332).

### Understanding doping stability of thin films *in vitro*

After revealing that there was an increased cell number on the surface of the FeCl_3_ doped DPP3T, we next sought to understand the doping stability in cell culture media. We first doped the polymer thin films, and then placed the samples within the media that the cells were grown in and kept within a humidified incubator for 48 hours to match the cell assay time.

After 48 hours in cell culture, it appeared that the doped films de-dope, evidenced by the re-emergence of the neutral absorption peak and loss in the polaron peaks (Suppl. 7, ESI†). As a positive control, a pristine undoped film was placed within the same conditions to see if the film itself was affect by being placed within cell culture media at 37 °C. There was no change to the overall shape of the incubator DPP3T thin film, however there was an alteration to the overall absorbance intensity, which may have been a result of adsorption of cell culture media components onto the surface of the film (Suppl. 7A, ESI†). DPP3T is insoluble within aqueous solvents, so it is unlikely that the polymer would be dissolved within the aqueous cell culture media, other than possibly some minor swelling. However, what is interesting, is that despite the de-doping within the samples, the cell number appears to remain unaffected by this in the FeCl_3_ doped DPP3T.

Recent literature has investigated the use of ionic exchange with dopants to reduce doping degradation on organic semiconducting films.^46^ We wanted to investigate if could prevent the degradation of our doped films over the 48 hour incubation period in cell culture media (Suppl. 8, ESI†). Using the same dopant concentrations along with lithium bis(trifluoromethanesylfonyl)imide (LiTFSI) in a ratio of 1 : 10 of dopant : LiTFSI in acetonitrile, we sought to investigate if this ion exchange, and ion ‘trapping’ of the dopant through counter ion switching would cause any difference in the doping kinetics and longevity of the doped films. We first uncovered that it is still possible to dope DPP3T within this ionic liquid using the same dopants, and interestingly that doping efficiency was altered within the same concentration of FeCl_3_ in comparison to using neat acetonitrile as the solvent. The polaron peak within the FeCl_3_:TFSI sample was increased in magnitude when using the same concentration of FeCl_3_. However, ion exchange doping was not able to stabilise the doping of the thin film over the assay period, and the thin films de-doped after 48 hours incubation within cell culture media. This is likely due to the complexity of cell culture media, which contains several buffers to stabilize the pH within cell culture.^47^

## Experimental

### Synthetic reagents

Solvents and compounds for synthesis were purchased from Sigma Aldrich, Fluorochem or Honeywell and used without further purification unless stated. For the doping of the DPP3T thin films, iron(iii) *p*-toluenesulfonate hexahydrate (Insight Biotechnology, UK), anhydrous iron(iii) chloride (FeCl_3_) (Insight Biotechnology) and tris(4-bromophenyl)ammoniumyl hexachloroantimonate (Magic Blue, Sigma Aldrich, UK), lithium bis(trifluoromethanesylfonyl)imide (LiTFSI) (Sigma Aldrich, UK) were used.

### Characterization of polymer

See ESI† on monomer and polymer synthesis.

### Spin coating of DPP3T

For the UV-Vis-NIR spectroscopy measurements DPP3T thin films were spin coated onto glass slides (1 × 1 cm) cleaned with soapy water, deionised water and then IPA by sonication for 15 minutes in each solvent, followed by UV-Ozone treatment for 2 mins using a (Ossila, UK). 30 μL of a 10 mg mL-1 DPP3T solution in chlorobenzene kept at 80 °C, was deposited onto the spinning substrate using dynamic spin coating on a vacuum free spin coater (Ossila, UK). The spin speed was 2000 rpm, and the time was 90 seconds to ensure dryness. Films were then dried and kept under vacuum prior to measurements. For cell culture experiments using the DPP3T thin films, films were spin coated and doped on top of borosilicate glass coverslips, which were used as received.

### Thin film doping

Dopants were dissolved in degassed acetonitrile within a series of concentrations, ranging from; iron tosylate hexahydrate (0.15 to 150 mM), anhydrous FeCl_3_ (0.002 to 30 mM), Magic Blue (0.06 to 0.6 mM). Dopants were chosen based on their doping strength, with iron tosylate hexahydrate possessing the weakest doping strength and Magic Blue possessing the highest within our study. Doping was conducted using spin coating. Briefly, 150 μL of dopant in acetonitrile was dropped onto the surface of the film and left for 60 seconds and then spun at 2000 rpm for 60 seconds. During the spin, 150 μL of neat acetonitrile was deposited to wash the surface of the film and remove the excess dopant. UV-Vis-NIR spectroscopy was conducted of both the washed samples and the unwashed sample to assess doping levels within the polymer.

For the ion exchange doping, LiTFSI was co-dissolved with the dopant at 10-fold greater concentration in degassed acetonitrile, *e.g.* for FeCl_3_ doping at 30 mM, we used LiTFSI at a concentration of 300 mM. The same method for doping was applied.

### UV-Vis-NIR spectroscopy

UV-Vis-NIR absorption spectroscopy of polymer films was performed with a Shimazdu UV-3600 Plus. Spectra were collected between 300–2500 nm with measurements taken every 1 nm.

### X-ray photoelectron spectroscopy (XPS)

All XPS measurements were carried out on a Thermo K-alpha spectrometer. A monochromatic Al K-Alpha X-ray source (1486.6 eV) with spot size 400 μm was used as the photon source and spectra were acquired in constant analyser energy mode. Survey spectra were recorded with a pass energy of 200 eV, and core level spectra were recorded with 50 eV pass energy. A dual beam flood gun was used to compensate the effects of sample charging. The samples were measured as received under a base pressure >5 × 10^−7^ mbar.

### Surface morphology of thin films

Atomic force microscopy (AFM) imaging was carried out using a Bruker Dimension Icon scanning probe microscope in peak-force tapping mode (using a Bruker ScanAsyst-Air silicon tip on nitride lever, *k* = 0.4 N m^−1^). All images were processed (background interpolation) and analysed (roughness parameters) using NanoScope Analysis.

### Water contact angle and surface energy measurements

2 μL of water was deposited onto the films and then an image was captured using Zelux 1.6-megapixel Thorlabs camera. Two images were captured for water for each slide. The contact angle was measured using ImageJ software with a contact angle plugin.^48^

For the surface energy calculations, a 2 μL droplet of diiodomethane was deposited and an image was captured using the same set up as above. The average contact angle was obtained from 2 measurements for both the water and diiodomethane droplets and the free surface energy was calculated using the Fowkes and Extended Fowkes Model.^49^ Diiodomethane was chosen as it has effectively no polar component to its surface tension, therefore the surface tension (50.8 mJ m^−2^) arises purely from the dispersive component. This allows for the calculation of the dispersive component to the surface energy of the thin film. Water was also chosen as its surface tension is also known (72.8 mJ m^−2^) and its dispersive and polar components are 21.8 mJ m^−2^ and 51.0 mJ m^−2^ respectively. Using these values and the calculated dispersive surface energy of the film, the polar component of the surface energy can be calculated. The surface energy is then the sum of the polar and the dispersive component.

### SCL1.4/F7 Schwann cell culture on materials

For assessment of the effects of pristine material and the doped films on cell behaviour, SCL1.4/F7s were seeded at a density of 15 000 cells per well on DPP3T coated glass coverslips in 24 well plates, allowed to settle for 3 hours and then placed within a humified cell culture incubator equipped with an Incucyte S3 (Satorius, UK). The Incucyte adherent cell-by-cell software module was used to quantify cell behaviour.

### Cell cytotoxicity evaluation

Live/dead analysis was conducted using calcein-AM and ethidium homodimer-1 for live and dead cells respectively using the manufacturer's protocol (Invitrogen, UK). Images were captured using Incucyte S3 green and red fluorescence detection channels. Percentage of live and dead cells was calculated using the total number of green and red objects.

### Statistical analysis

For viability and proliferation of Schwann cells interfaced with the materials, normality was assessed using a Shapiro–Wilk test. One-way ANOVA analysis was used and Dunnett's multiple comparisons or Tukey's multiple comparisons *post hoc* tests were used to determine whether there were statistically significant differences between the experiment groups. A probability (*p*) value of less than 0.05 was considered to indicate the presence of a significant difference. Data presentation and statistical analysis was performed with GraphPad Prism 9.0.0. Data are represented as mean ± standard error.

## Conclusions

Within this experimental report, we investigated the effects of molecular doping a widely investigated organic semiconducting polymer for bioelectronic devices, DPP3T with two separate p-type dopants and evaluated the impact on Schwann cell behaviour. We demonstrated successful doping of the polymer thin films in air, and showed that doping offers a facile and cheap way to successfully modulate cell behaviour on the surfaces of the thin films by changing the electronic and surface properties of the polymers. By considering molecular doping in conjunction with biomaterial requirements, our approach does not only hold great promise to optimise the electronic properties of the organic semiconductor, but at the same time alters the surface properties of the film to facilitate cellular interactions. Controlling and modulating the material–cell interface will be a key requirement in the development and translation of organic semiconducting materials towards integrated and directly interfaced biomedical applications.

## Author contributions

RPT, PGF, MMW. contributed to conception, design, data acquisition (UV-vis spectroscopy, film doping, AFM, Schwann cell work) and interpretation, drafted, and critically revised the article. AD and RP contributed to data acquisition (XPS) and interpretation, drafted, and critically revised the article. JBP, ABT & BCS contributed to conception, drafted, and critically revised the article. All authors gave their final approval and agree to be accountable for all aspects of the work.

## Conflicts of interest

There are no conflicts to declare.

## Supplementary Material

TC-011-D3TC01148H-s001
