# Supplementary material for: Improving the biological interfacing capability of diketopyrrolopyrrole polymers via p-type doping
Source: J Mater Chem C Mater. 2023 May 11;11(21):6943–50. doi: 10.1039/d3tc01148h (PMC10233798; doi:10.1039/d3tc01148h)
Supplement: TC-011-D3TC01148H-s001 [file TC-011-D3TC01148H-s001.pdf]

Electronic Supplementary Material (ESI) for Journal of Materials Chemistry C.
This journal is © The Royal Society of Chemistry 2023

Supplementary Information

Improving the biological interfacing capability of diketopyrrolopyrrole polymers via p-type doping

*Ryan P. Trueman, Peter Gilhooly Finn, Megan M. Westwood, Avishek Dey, Robert Palgrave, Alethea Tabor, James B. Phillips, Bob C. Schroeder**

S1. Synthetic procedures

Solvents and compounds were purchased from Sigma Aldrich, Fluorochem or Honeywell. 2,5-bis(trimethylstannyl)thiophene was recrystallised twice from acetonitrile and bis(dibenzylideneacetone)palladium (0) was recrystallised according to a literature procedure[2] prior to use. All other compounds were used without further purification. Synthesised compounds were made using standard Schlenk line techniques under a positive pressure of N_2. All glassware was dried at 120 °C overnight. Flash column chromatography was carried out using C60 silica gel and thin layer chromatography (TLC) analyses performed on silica gel 60 F254 plates (developed using a UV lamp at λ= 254 nm). ^{1}H and ^{13}C nuclear magnetic resonance (NMR) experiments were performed using a Bruker Avance 500 MHz spectrometer. Spectra were processed and analysed using MestreNova software, with all chemical shifts (δ) being reported relative to the solvent peak ($CDCl_3$: δ 7.26, 77.2 ppm; DMSO-d_6: δ 2.50, 39.5 ppm). Mass spectrometry was carried out on Waters UPLC-SQD mass spectrometer. Number average (M_n) and weight average (M_w) molecular weight values were determined using Size Exclusion GPC was carried out using Shimadzu Prominence GPC system, comprised of a SIL-20A auto sampler, LC-20AT liquid chromatograph, CTO-20A column oven, RID-20A refractive index detector and an SPD- 20A UV-vis detector. HPLC grade chloroform stabilised with amylene was purchased from Acros Organics. Analysis was carried out on Shimadzu's LabSolutions software. Atomic force microscopy (AFM) imaging was carried out using a Bruker Dimension Icon scanning probe microscope in peak-force tapping mode (using a Bruker ScanAsyst-Air silicon tip on nitride lever, k = 0.4 N/m). All images were processed (background interpolation) and analysed (roughness parameters) using NanoScope Analysis.

S2. Experimental procedures

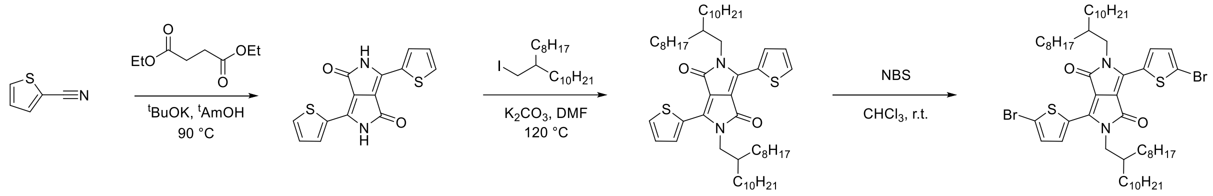

*Scheme 1: Synthetic route to monomer **3**, used in Stille coupling reaction to form DPP3T.*

3,6-bis(thiophen-2-yl)pyrrolo[3,4-*c*]pyrrolo-1,4(2*H*,5*H*)-dione (1)

Compounds 1, 2, 3 and the polymer DPP3T were synthesized according to previously published procedures[1]. 2-thienocarbonitrile (21.3 mL, 229 mmol) and potassium tert-butoxide (51.4 g, 458 mmol) were dissolved in tert-amyl alcohol (200 mL) in a 2-necked round bottomed flask and heated to 60 °C. Diethyl succinate ester (15.2 mL, 91.6 mmol) dissolved in tert-amyl alcohol (30 mL) was added dropwise over 1 h. The reaction mixture was stirred continuously at 90 °C for 12 hours before cooling to room temperature and neutralized to pH = 7.0 by addition of acetic acid. The precipitate was filtered, washed with H_2O (2x 200 mL) and methanol (2x 200 mL) and to give a dark red solid (14.2 g, 52%) that was dried at 40 ˚C under vacuum. ^1H NMR (500 MHz, DMSO) δ 11.23 (s, 2H), 8.21 (dd, *J* = 3.8, 1.2 Hz, 2H), 7.95 (dd, *J* = 5.0, 1.1 Hz, 2H), 7.30 (dd, *J* = 5.0, 3.8 Hz, 2H).

3,6-bis(thiophen-2-yl)-2,5-bis(2-octyldodecyl)pyrrolo[3,4-*c*]-1,4(2*H*,5*H*)-dione (2)

1 (5.54 g, 12.4 mmol) and potassium carbonate (6.00 g, 43.4 mmol) were added to a dry 2-necked round bottomed flask before addition of DMF (120 mL). The reaction mixture was heated to 65 °C and stirred over 1 hour. 1-Iodo-2-octyldodecane (15.3 g, 37.4 mmol) was then added dropwise over 30 minutes and the reaction mixture was stirred at 120 °C overnight before cooling to room temperature. The solvent was removed *in vacuo* and the organics extracted with CH_2Cl_2 (300 mL). Organic layer was washed with H_2O (3x 250 mL), brine (250 mL) and dried ($MgSO_4$). After filtration, the solvent was removed *in vacuo* and the crude purple product was purified by flash column chromatography (eluting with EtOAc:Hexane (1:9), v/v) to yield **2** (3.25 g, 21 %) as a waxy purple solid that was dried under vacuum. R_f = 0.58. ^1H NMR (500 MHz, $CDCl_3$) δ 8.87 (d, *J* = 3.9 Hz, 2H), 7.62 (d, *J* = 5.0 Hz, 2H), 7.27 (s, 1H), 4.02 (d, *J* = 7.7 Hz, 4H), 1.91 (d, *J* = 9.4 Hz, 2H), 1.55 (s, 4H), 1.32 – 1.17 (m, 66H), 0.86 (q, *J* = 7.2 Hz, 12H).

3,6-Bis(5-bromothiophen-2-yl)-2,5-bis(2-octyldodecyl)pyrrolo[3,4-*c*]-1,4(2*H*,5*H*)-dione (3)

2 (3.00 g, 3.48 mmol) was added to a dried 2-necked round bottom flask and dissolved in $CHCl_3$ (100 mL) under stirring. The flask was covered in foil to exclude light. *N*-bromosuccinimide (1.30 g, 7.31 mmol) was added and the reaction mixture stirred overnight. The organics were extracted with $CHCl_3$ and washed with H_2O (2 x 75 mL), brine (75 mL) and dried ($MgSO_4$). After filtration, the organic layer was concentrated under vacuum and purified *via* flash column chromatography (eluting with chloroform:hexane (1:4, v/v)) to yield **3** (3.10 g, 81 %) as a dark purple waxy solid that was dried under vacuum. R_f = 0.21. ^{1}H NMR (500 MHz, $CDCl_3$) δ 8.62 (d, *J* = 4.2 Hz, 2H), 7.22 (d, *J* = 4.2 Hz, 2H), 3.92 (d, *J* = 7.7 Hz, 4H), 1.87 (d, *J* = 7.8 Hz, 2H), 1.31 – 1.19 (m, 64H), 0.87 (m, *J* = 6.9 Hz, 12H).

Polymer synthesis (DPP-3T)

3 (0.1049 g, 0.1029 mmol), 2,5-bis(trimethylstannyl)thiophene (0.0422 g, 0.1029 mmol), bis(dibenzylideneacetone)palladium (0) (2.40 mg, 2 mol%) and tri(*o*-tolyl)phosphine (5.20 mg, 8 mol%) were added to a dried microwave vial. Chlorobenzene (2 mL) was added and the solution degassed over 10 minutes before capping. The vial was transferred to a microwave reactor and heated stepwise 100 °C (2 min), 120 °C (2 min), 140 ° C (2 min), 160 °C (5 min) and 180 °C (40 mins). To endcap, the reaction was cooled to 55 °C and trimethyl(phenyl)tin (8 µL, 0.0431 mmol) added. The vial was resubmitted to the microwave and heated under stirring at 160 °C for 5 minutes. This step was repeated with bromobenzene (9 µL, 0.0862 mmol). When cooled, the vial was uncapped the reaction mixture extracted with $CHCl_3$ (20 mL). Sodium thiodicarbamate was added and the solution heated to 60 °C under N_2 (2 h). Deionised water (60 mL) was added and the solution stirred at 60 °C overnight. After cooling to room temperature, the polymer was extracted with $CHCl_3$ (100 mL) and washed with deionised water (3x 100 mL). The organic solvent was concentrated under vacuum and the polymer precipitated into an excess of stirring MeOH. The precipitate was filtered into a glass fibre thimble and the polymer purified *via* Soxhlet extraction with MeOH, acetone and hexane (24 h, each) before extracting with $CHCl_3$. The polymer was precipitated into stirring MeOH and collected by vacuum filtration to yield **DPP3T** as a dark blue solid (0.136 g, 66%). M_n = 79 kDa, Đ = 2.0.

S3. NMR Spectra

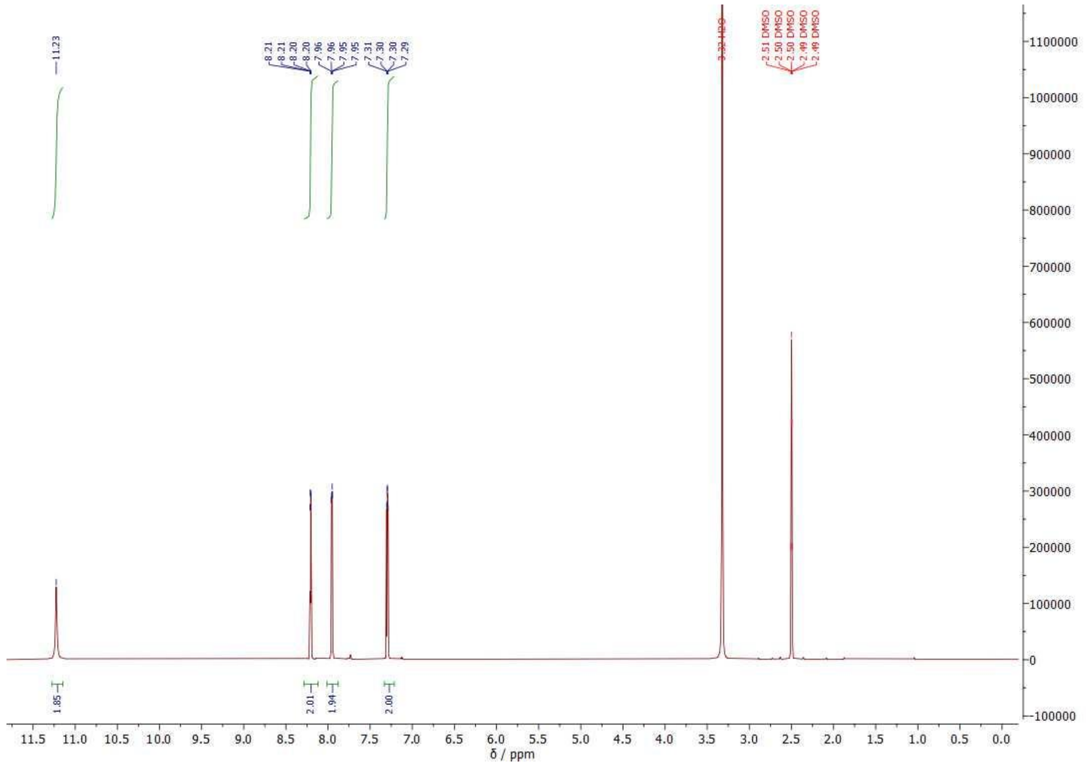

*Figure S1: Proton NMR spectra of compound **1** recorded in DMSO-d$_6$ (500 MHz).*

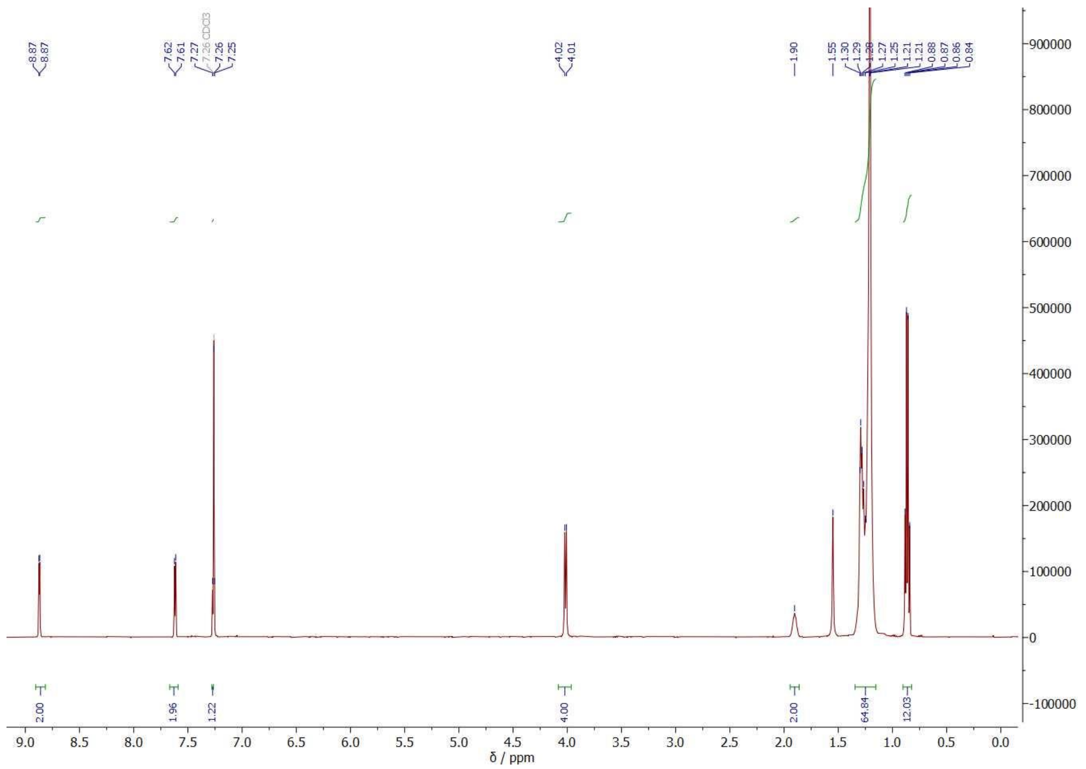

*Figure S2: Proton NMR spectra of compound **2** recorded in $CDCl_3$ (500 MHz).*

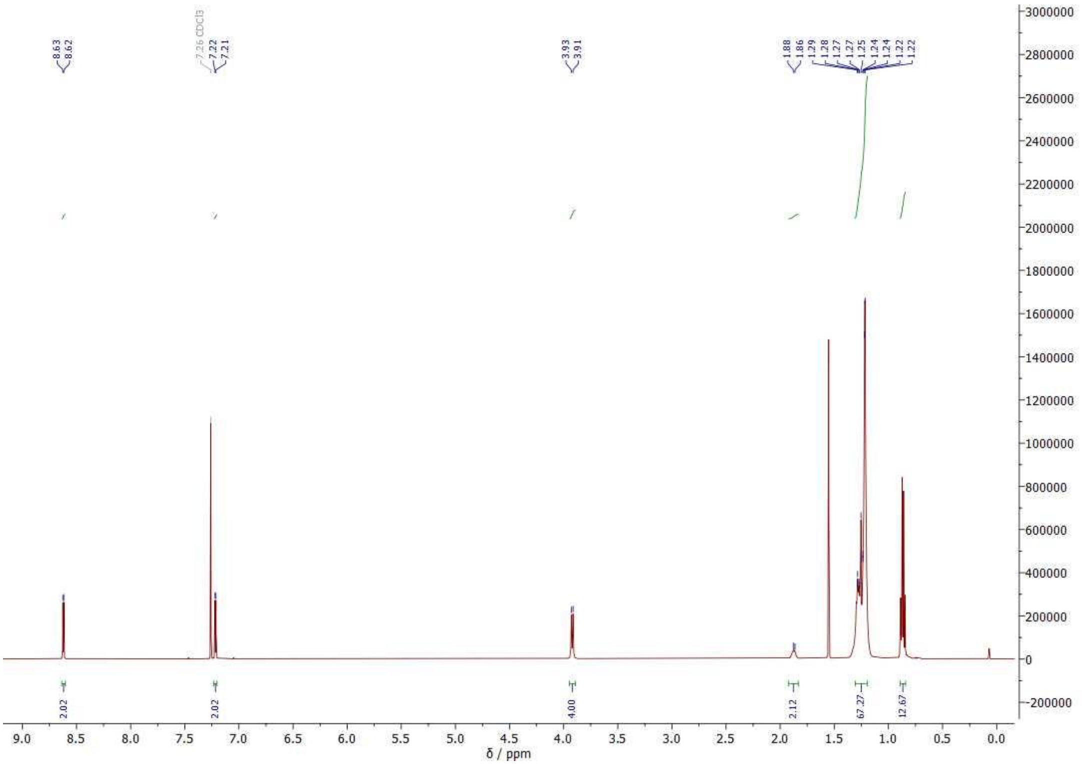

*Figure S3: Proton NMR spectra of compound **3** recorded in $CDCl_3$ (500 MHz).*

S4. Supplementary Results & Discussion

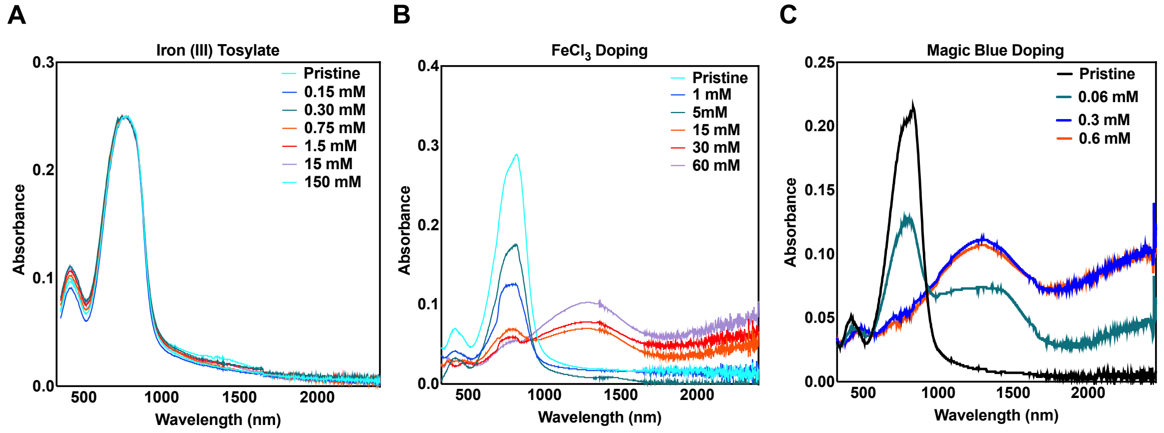

Suppl. 1 UV-vis spectra of DPP3T doped with increasing concentrations of A) iron tosylate hexahydrate, B) anhydrous FeCl3, C) Magic Blue.

Suppl. 1 reports the UV-vis-NIR spectra of DPP3T doped with the 3 p-type dopants employed within this study. Firstly, Iron (III) tosylate hexahydrate was used to attempt to dope DPP3T thin films, however no alteration of the absorbance features was seen, indicating that no electron transfer occurred from the film to the p-type dopants. Secondly, $FeCl_3$, a commonly

used dopant within organic semiconducting literature for high performance devices was used. $FeCl_3$ successfully doped the DPP3T film in air, however the concentration was higher than that usually seen within the literature. $FeCl_3$ is cytotoxic, and therefore once we had seen successful doping, we did not chose to increase the dose of $FeCl_3$ any higher. Lastly, Magic Blue, a less common p-type dopant was utilized to dope DPP3T. Magic Blue required a 100 fold lower concentration to completely saturate the primary absorbance band of neutral DPP3T, and it is this concentration that was taken forward to the cell study.

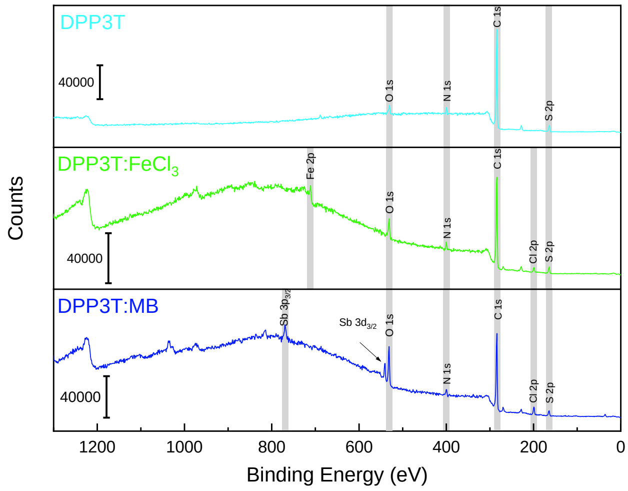

Suppl. 2. XPS survey scan of the surface of DPP3T, DPP3T:$FeCl_3$ and DPP3T:MB films. Grey lines highlight the regions of the important peaks.

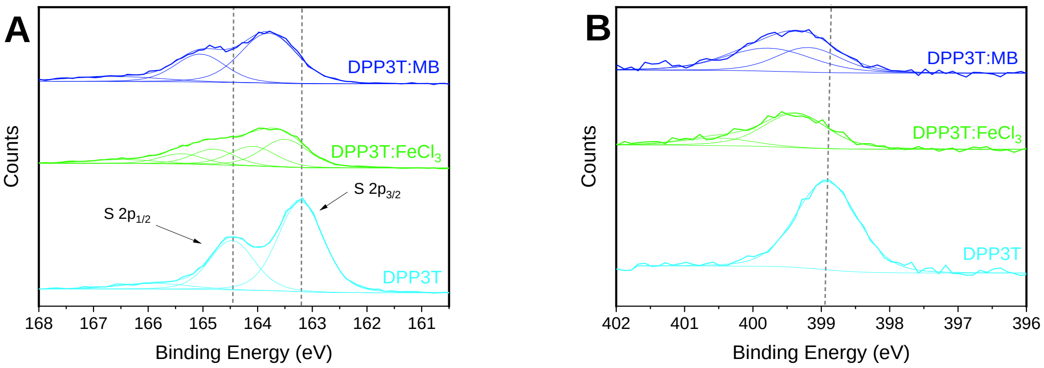

Suppl. 3. Zoomed in spectra of A) S 2p and B) N 1s peaks obtained from the surface of DPP3T, DPP3t:FeCl3 and DPP3T:MB films. The dotted line highlights to the reader where the S 2p3/2/2p1/2 and N 1s peak is of the neutral DPP3T for Figure A and B respectively.

We then studied the composition of elements on the surface of the neutral and doped films using XPS (**Suppl. 2 & 3)**. In the survey scan, the neutral DPP3T films show peaks associated to oxygen, nitrogen, carbon and sulphur arising from the polymer. $FeCl_3$ doped films showed the same peaks as the neutral films however, peaks for Fe 2p and Cl 2p are also present indicating $FeCl_3$ is on the surface. Magic Blue doped films also tell a similar story, but the new peaks arise from Sb $3d_{3/2}$ around 540 eV and Cl 2p. The Sb $3d_{5/2}$ overlaps with O 1s. Interestingly we observed no bromine present in the survey scan of the Magic Blue doped films indicating that the tris(4-bromophenyl)ammoniumyl counter cation is no longer present possibly due to the washing step[3]. We also found that the S 2p and N 1s peaks for the MB and $FeCl_3$ doped DPP3T films shift to higher binding energies by around 2 eV compared with the undoped film, which has been associated to oxidation of the polymer in the literature[4].

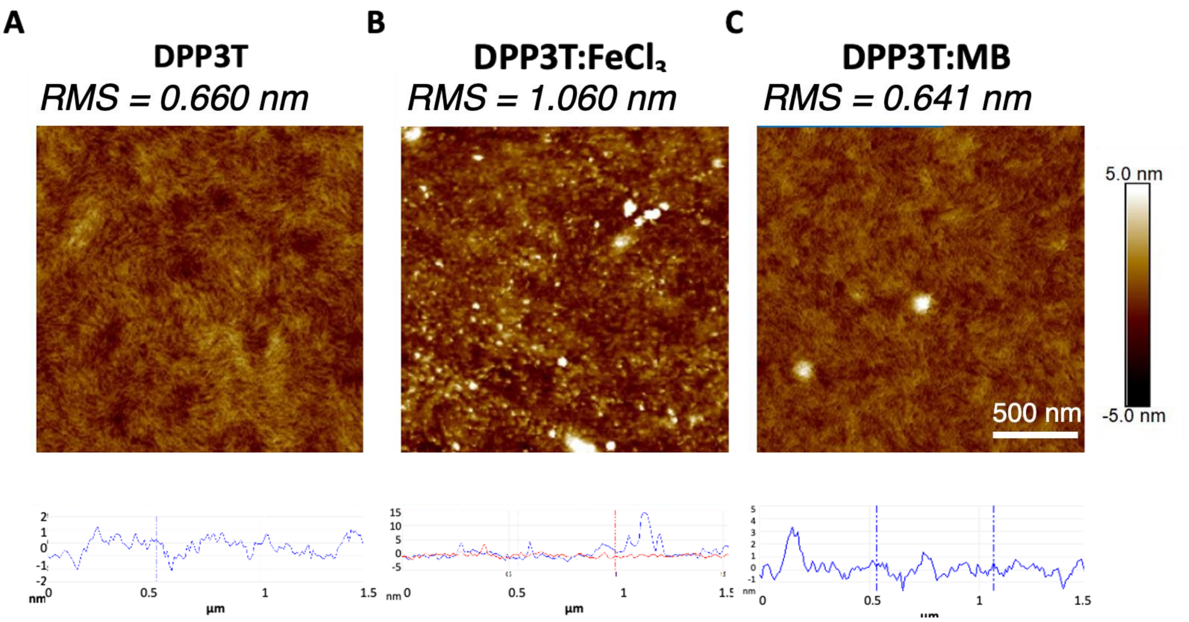

Suppl. 4 AFM images of the DPP3T (A) pristine film surface, (B) doped with $FeCl_3$ and (C) doped with Magic Blue.

Suppl. 4 shows the AFM images captured of the doped films, with height profiles. DPP3T doped with $FeCl_3$ displays significantly rougher surface with features of increased height in comparison to the pristine film.

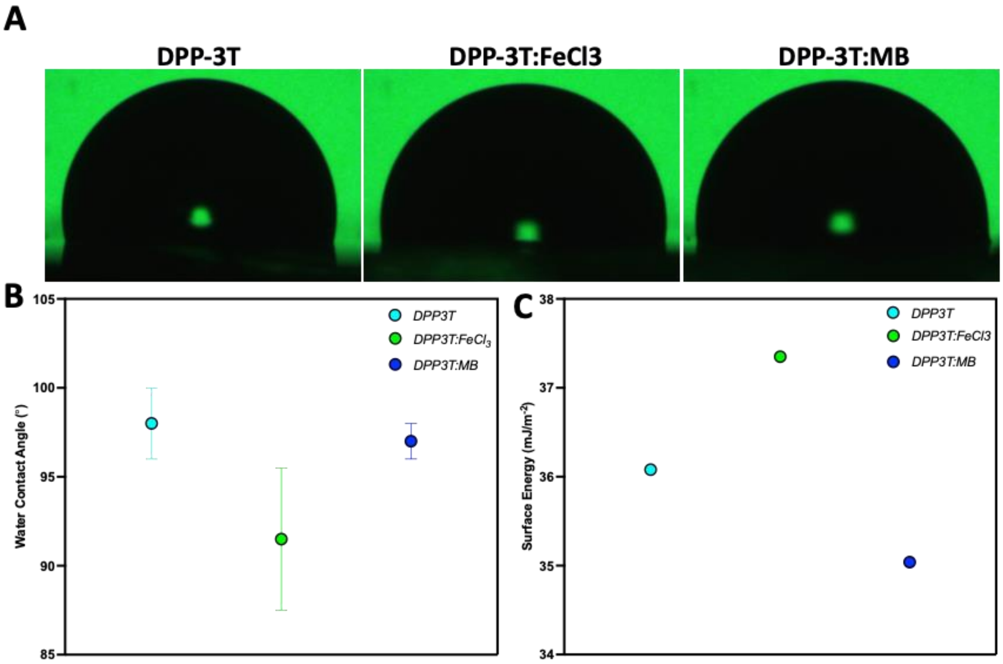

Suppl. 5 Water contact angle measurements of the DPP3T Doped surfaces. A) Photographs of a 2 µL droplet of water on the surfaces of the films, either pristine, or doped with either FeCl3 or Magic Blue. B) Quantification of water contact angle from images. Data presented as average of two measurements with error bars (Mean +/- standard error) C) Surface energy calculated using the Fowkes and Extended Fowkes Model[5].

Table 1: Water contact angle measurements for the pristine and doped DPP3T thin films

Sample	**Water Contact Angle (°)**		**Diiodomethane Contact Angle (°)**	
	**Droplet 1**	**Droplet 2**	**Droplet 1**	**Droplet 2**
DPP3T	100	96	58	56
DPP3T:$FeCl_3$	95	95	60	58
DPP3T:MB	101	101	53	54

Table 2: Surface energy measurements taken from the pristine and doped DPP3T thin films.

Sample	**Surface Energy (mJ m^{-2})**		
	**Dispersive Component**	**Polar Component**	**Total**
DPP3T	30.3	5.8	36.1
DPP3T:$FeCl_3$	29.2	8.2	37.3
DPP3T:MB	32.3	2.7	35.0

To understand the hydrophilicity of the doped polymer films in comparison to the pristine, water contact angle measurements and surface energy calculations were performed (**Suppl. 5**). The contact angle of 2 water droplets were measured for each film and on average the $FeCl_3$ doped films exhibited a lower contact angle (95°) than DPP3T (98°) and DPP3T:MB (101°) (**Table 1**). Measuring the contact angle of 2 diiodomethane droplets as well for each film, we could then calculate the surface energy of the films (**Table 2**). Although we found that the associated energies are very similar (~36 mJ m^{-2}) and probably within the error of the measurement, $FeCl_3$ doped films do exhibit the highest surface energy and the lowest water contact angle, indicating a more hydrophilic surface which may influence cell adhesion properties[6, 7].

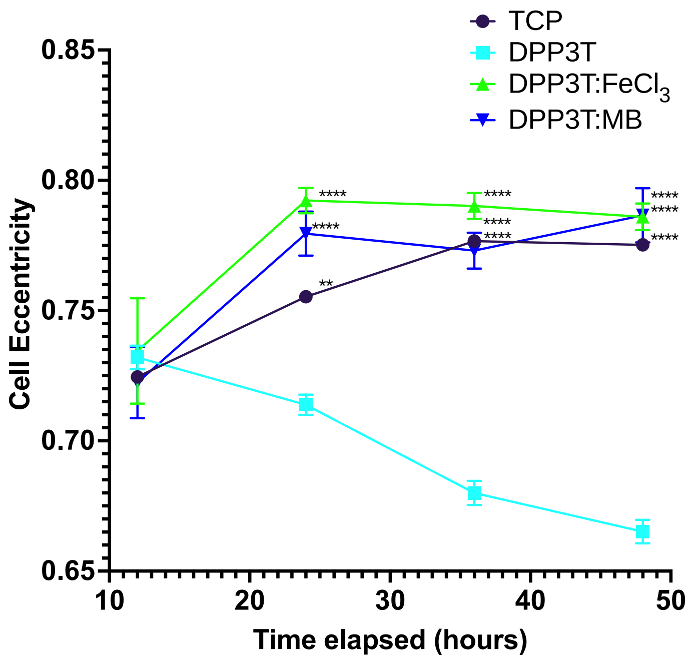

*Suppl. 6 Cell eccentricity over a 48-hour time point. Values range between 0 and 1, with 0 being a perfect circle. N=6 independent DPP3T films per experimental group, with 9 images per film, per time point analysed for the cell count per image mean. Data presented as mean +/- SEM. C) Stats = Two-Way ANOVA ($p = <0.0001$) with Dunnetts multiple comparisons test run post hoc in comparison to the pristine DPP3T film.. $p < 0.05$ denotes significance, with * = $p < 0.05$, ** = $p < 0.01$, *** = $p < 0.001$ **** = $p < 0.0001$ denoted on the graphs.*

Within the pristine DPP3T thin film and two doped films, the cells possessed a visually different morphology (**Suppl. 6**). The change in morphology was quantified using advanced image analysis using the Sartorius Incucyte analysis of adherent cell-by-cell from the phase contrast images. This allows assessment of cell shape, in particular eccentricity, with values ranging from 0 , representing a perfect circle, to 1, constituting an extremely elongated shape. This information provided qualitative information which can be combined with information about cell size to give insights into the overall adhesion of the cells on top of the respective surface. There was a significant difference in cell shape for all the groups in comparison to the pristine DPP3T group.

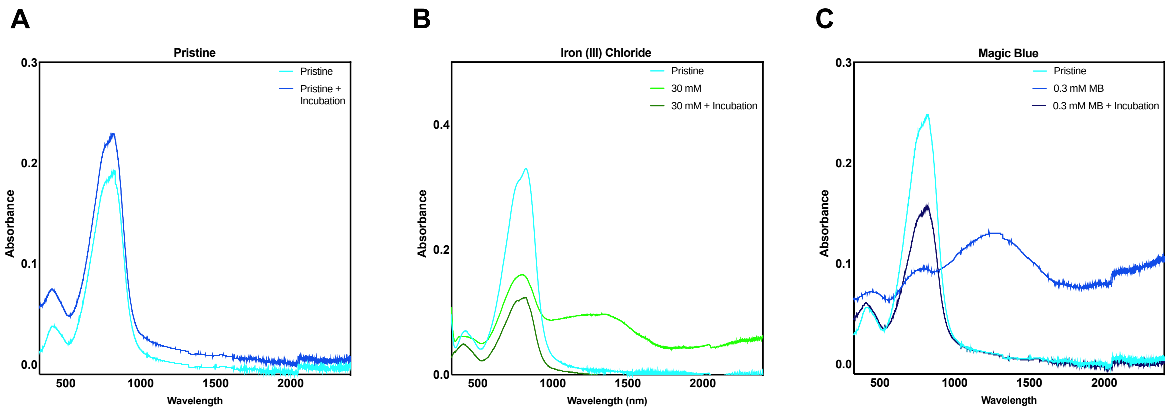

Suppl. 7 Thin film UV-vis-NIR absorbance quantification of doping levels after incubation within cell culture media. A) Pristine DPP3T thin film B) DPP3T thin film doped with 30 mM of FeCl3 C) DPP3T thin film doped with 0.3 mM of Magic Blue (MB). All samples were incubated for 48 hours within Dulbecco's Modified Eagle Medium (high glucose) for 48 hours within a cell culture incubator. The strong change in absorbance at around 2000 nm is due to a lamp change.

After 48 hours in cell culture, it appeared that the doped films de-dope, evidenced by the re-emergence of the neutral absorption peak and loss in the polaron peaks (**Suppl. 7**). As a positive control, a pristine undoped film was placed within the same conditions to see if the film itself was affect by being placed within cell culture media at 37 °C. There was no change to the overall shape of the incubator DPP3T thin film, however there was an alteration to the overall absorbance intensity, which may have been a result of adsorption of cell culture media components onto the surface of the film (**Suppl. 7A**). DPP3T is insoluble within aqueous solvents, so it is unlikely that the polymer would be dissolved within the aqueous cell culture media, other than possibly some minor swelling. However, what is interesting, is that despite the de-doping within the samples, the cell number appears to remain unaffected by this in the $FeCl_3$ doped DPP3T.

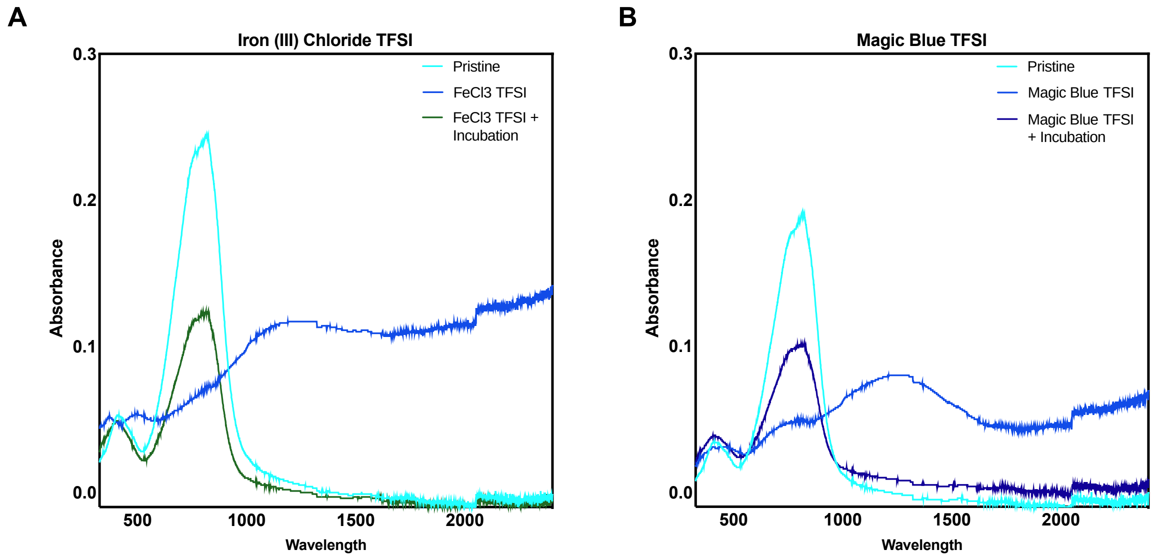

Suppl. 8 Thin film UV-vis-NIR Spectra of $FeCl_3$ and Magic Blue doped DPP3T thin films in ionic liquid (acetonitrile + TFSI). A) DPP3T doped thin film with 30 mM of FeCl3 in a 300 mM solution of LiTFSI in acetonitrile. B) DPP3T doped thin film with 0.3 mM Magic Blue in a 30 mM solution of LiTFSI in acetonitrile. The strong change in absorbance at around 2000 nm is due to a lamp change.

Ion-exchange doping was investigated, to assess if degradation of our doped films over the 48-hour incubation period in cell culture media could be prevented. (**Suppl. 8**). Using the same dopant concentrations along with salt lithium bis(trifluoromethanesylfonyl)imide (LiTFSI) in a ratio of 1:10 of dopant:LiTFSI in acetonitrile, we sought to investigate if this ion exchange, and ion 'trapping' of the dopant through counter ion switching would cause any difference in the doping kinetics and longevity of the doped films. We first uncovered that it is still possible to dope DPP3T within this ionic liquid using the same dopants according to the UV-vis-NIR data, and interesting that doping efficiency was altered within the $FeCl_3$ sample in comparison to using neat acetonitrile as the solvent. However, we uncovered that this was not the case, and the thin films de-doped after 48 hours incubation.

S4. References

1. Zhang, X. et al. Molecular Packing of High-Mobility Diketo Pyrrolo-Pyrrole Polymer Semiconductors with Branched Alkyl Side Chains. *Journal of the American Chemical Society* **133**, 15073-15084 (2011).

2. Zalesskiy, S.S. & Ananikov, V.P. Pd2(dba)3 as a Precursor of Soluble Metal Complexes and Nanoparticles: Determination of Palladium Active Species for Catalysis and Synthesis. *Organometallics* **31**, 2302-2309 (2012).

3. Yamashita, Y. et al. Supramolecular cocrystals built through redox-triggered ion intercalation in π-conjugated polymers. *Communications Materials* **2**, 45 (2021).

4. Jacobs, I.E. et al. Structural and Dynamic Disorder, Not Ionic Trapping, Controls Charge Transport in Highly Doped Conducting Polymers. *Journal of the American Chemical Society* **144**, 3005-3019 (2022).

5. Fowkes, F.M. ATTRACTIVE FORCES AT INTERFACES. *Industrial & Engineering Chemistry* **56**, 40-52 (1964).

6. Hallab, N.J., Bundy, K.J., O'Connor, K., Moses, R.L. & Jacobs, J.J. Evaluation of metallic and polymeric biomaterial surface energy and surface roughness characteristics for directed cell adhesion. *Tissue engineering* **7**, 55-71 (2001).

7. Majhy, B., Priyadarshini, P. & Sen, A. Effect of surface energy and roughness on cell adhesion and growth–facile surface modification for enhanced cell culture. *RSC advances* **11**, 15467-15476 (2021).
